# Convergent Loss of Awn in Two Cultivated Rice Species *Oryza sativa* and *Oryza glaberrima* Is Caused by Mutations in Different Loci

**DOI:** 10.1534/g3.115.020834

**Published:** 2015-09-02

**Authors:** Tomoyuki Furuta, Norio Komeda, Kenji Asano, Kanako Uehara, Rico Gamuyao, Rosalyn B. Angeles-Shim, Keisuke Nagai, Kazuyuki Doi, Diane R. Wang, Hideshi Yasui, Atsushi Yoshimura, Jianzhong Wu, Susan R. McCouch, Motoyuki Ashikari

**Affiliations:** * Bioscience and Biotechnology Center, Nagoya University, Furo-cho, Chikusa, Nagoya, Aichi 464-8601, Japan; † Department of Plant Breeding and Genetics, Cornell University, Ithaca, New York 14853-1901; ‡ Plant Breeding Laboratory, Kyushu University, Higashi-ku, Fukuoka 812-8581, Japan; § National Institute of Agrobiological Sciences, Tsukuba, Ibaraki 305-8634, Japan

**Keywords:** awn, domestication, Asian rice, African rice, CSSLs

## Abstract

A long awn is one of the distinct morphological features of wild rice species. This organ is thought to aid in seed dispersal and prevent predation by animals. Most cultivated varieties of *Oryza sativa* and *Oryza glaberrima*, however, have lost the ability to form long awns. The causal genetic factors responsible for the loss of awn in these two rice species remain largely unknown. Here, we evaluated three sets of chromosome segment substitution lines (CSSLs) in a common *O. sativa* genetic background (cv. Koshihikari) that harbor genomic fragments from *Oryza nivara*, *Oryza rufipogon*, and *Oryza glaberrima* donors. Phenotypic analyses of these libraries revealed the existence of three genes, *Regulator of Awn Elongation 1 (RAE1)*, *RAE2*, and *RAE3*, involved in the loss of long awns in cultivated rice. Donor segments at two of these genes, *RAE1* and *RAE2*, induced long awn formation in the CSSLs whereas an *O. sativa* segment at *RAE3* induced long awn formation in *O. glaberrima*. These results suggest that the two cultivated rice species, *O. sativa* and *O. glaberrima*, have taken independent paths to become awnless.

The awn, a typical feature of Poaceae, is a needle-like organ extending from the tip of the lemma and is considered to be a modified leaf blade (Dahlgren *et al.* 1985). This bristly, barbed extension of the spikelet facilitates seed dispersal by attaching the seed to animal fur and deters seed predation by birds and mammals (Grundbacher 1963). In some cases, such as in wild tetraploid wheat, the movement of awns may even propel the seed into soil (Elbaum *et al.* 2007; Elbaum *et al.* 2008). Despite the important roles of grass awns under wild conditions, long and barbed awns hinder manual harvesting under agricultural conditions and have largely been avoided during artificial selection of rice by humans (Takahashi 1955). In contrast to barley awns, which are capable of photosynthesis during grain-filling, rice awns lack chlorenchyma and cannot contribute to photosynthesis (Grundbacher 1963; Kjack and Witters 1974; Takahashi *et al.* 1986; Tatsumi and Kawano 1972). Consistent with that observation, removal of awns has been shown to have a negligible effect on rice grain maturation (Tsudamori 1933) and, consequently, cultivated rice varieties may have become awnless to enhance ease of harvest without adverse effects on yield.

The genus *Oryza* has two independently domesticated species: cultivated Asian rice (*Oryza sativa*) and cultivated African rice (*Oryza glaberrima*) (Khush 1997). Domestication of *O. sativa* from its wild progenitor, *Oryza rufipogon*, is thought to have started ∼8000 years ago, and there is evidence of a polyphyletic origin of *O. sativa* (Yamanaka *et al.* 2002; Cheng *et al.* 2003; Fuller *et al.* 2010; Huang *et al.* 2012). In contrast to its Asian cultivated counterpart, *O. glaberrima* was domesticated from *Oryza barthii* in West Africa more recently, ∼3500 years ago (Linares 2002; Li *et al.* 2011). Despite the independent domestication histories of *O. sativa* and *O. glaberrima*, most varieties of both species are awnless, whereas their ancestral species, *O. rufipogon* and *O. barthii*, possess long awns (Chang *et al.* 1977). Past genetic studies have identified multiple awn-related quantitative trait loci (QTL) in rice (Sato *et al.* 1996; Xiong *et al.* 1999; Lorieux *et al.* 2000; Thomson *et al.* 2003; Yoshimura *et al.* 2010); however, only one gene has been cloned. This gene, named *An-1*, is a bHLH transcription factor located on chromosome 4, which was identified using an interspecific cross between *O. rufipogon* and *O. sativa* (Luo *et al.* 2013). The study showed that the *O. sativa* allele of *An-1* has lost its prolonged expression at the distal end of the lemma compared to the *O. rufipogon* allele. This expression change confers the awnless phenotype in grains of *O. sativa*. While a recent mutant study revealed two additional developmental genes, *DL* and *OsETT2*, that also affect awn morphology (Toriba and Hirano 2014), the regulatory mechanism of long awn formation is still largely unknown and the question of how *O. sativa* and *O. glaberrima* lost their awns remains.

In this study, three sets of chromosome segment substitution lines (CSSLs) in a common *O. sativa* genetic background were used to identify three long awn-inducing loci. The three loci, *Regulator of Awn Elongation 1 (RAE1)*, *RAE2*, and *RAE3*, may be involved in the loss of long awns during the domestication of *O. sativa* and *O. glaberrima*. Our data suggest that *O. sativa* lost the function of *RAE1* and *RAE2*, whereas *O. glaberrima* achieved an awnless phenotype through mutation(s) in *RAE3* while maintaining functional alleles at both *RAE1* and *RAE2*. This report is the first account of two closely related crop species converging on a single morphological feature through mutations in different genes.

## Materials and Methods

### Plant materials

*O. sativa* ssp. *japonica* cv. Koshihikari (hereinafter referred to as Koshihikari), *O. glaberrima* Acc IRGC104038, and three sets of **c**hromosome **s**egment **s**ubstitution **l**ines (CSSLs) were used for phenotypic analyses to identify loci that control long awn formation. The three CSSL populations (WBSLs, RSLs, and GLSLs) share Koshihikari as the recurrent parent but differ in their donor parents: WBSLs contain *Oryza nivara* Acc W0054 donor introgressions; RSLs contain *O. rufipogon* Acc W0106 donor introgressions (Furuta *et al.* 2014); and GLSLs contain *O. glaberrima* Acc IRGC104038 donor introgressions (Shim *et al.* 2010). These plant materials were grown either in the greenhouses of the Laboratory of Plant Molecular Biosystems or under natural conditions in the research field of Nagoya University, Togo, Aichi, Japan. Seedlings were first grown in the greenhouse for 30 d and then transplanted in the field.

### Measurement of yield related traits and awn frequency per panicle

Panicles of the CSSLs were harvested after seed maturation. We measured the following yield-related and awn traits: panicle length; number of primary branches; number of seeds per panicle; seed length; seed width; and awn frequency per panicle. Any seed with an awn longer than 3 mm (measured by a ruler) was considered awned. Awn frequency per panicle was calculated as the number of awned seeds per panicle divided by the total number of seeds per panicle. Seed length and width were measured using a scanned image analyzing software called SmartGrain (Tanabata *et al.* 2012).

### Linkage analysis and fine mapping of *RAE1* and *RAE3*

To map *RAE1*, ∼8000 F_2_ plants were produced by crossing GLSL-13 with Koshihikari. For *RAE3*, we used the progenies of 54 BC_4_F_1_ derived from a cross between *O. glaberrima* Acc IRGC104038 as the recurrent female parent and *O. sativa* cv. Taichung65 as the donor male parent. To develop the BC_4_F_1_ populations, F_1_ plants derived from a cross between *O. glaberrima* and Taichung65 were backcrossed successively to *O. glaberrima* four times. The 54 BC_4_F_2_ populations comprising approximately 100 plants each were subjected to the linkage analysis between awn formation and the genotypes of the mapping population. Each mapping population was genotyped using SSR and insertion/deletion (indel)-based markers developed to target the putative gene location (see Supporting Information, Table S1 for primer sequences). Genomic DNA from the mapping population was extracted using the TPS method (Hattori *et al.* 2007). SSRs and indel markers were amplified using standard PCR protocols and run on a 3% agarose gels containing ethidium bromide.

### Sequence analysis of *RAE1*

A BAC clone library for *O. glaberrima* Acc IRGC104038 was provided by Honda Research Institute (HRI) in Kisarazu, Chiba, Japan. The BAC clone (HWC026-A20) carrying the *RAE1* candidate locus was screened from the BAC clone library using the flanking markers previously used for fine mapping of *RAE1*. Sequencing of the BAC clone was performed by shotgun sequence method using an ABI3700 capillary DNA sequencer (Applied Biosystems). Base-calling and assembly were performed using Sequencher sequence analysis software (HITACHI SOFT). Comparative analysis between sequences within the candidate region for *RAE1* derived from the BAC clone and the corresponding sequences in Nipponbare [**R**ice **A**nnotation **P**roject **D**ata**b**ase, (RAP-DB); IRGSP-1.0] was performed using ClustalW ver. 2.0 set at the default settings (Larkin *et al.* 2007).

### Transformation constructs for complementation and overexpression

To develop a subclone for the complementation test of *RAE1*, the DNA of BAC clone HWC026-A20 was partially digested with *Hind*III to generate genomic fragments containing the *RAE1* candidate locus. The binary vector, *pYLTAC7* (Liu *et al.* 1999), was digested with *Hind*III and dephosphorylated by calf intestine alkaline phosphatase (CIAP) treatment. The BAC DNA fragments were ligated to the digested *pYLTAC7* using Takara DNA Ligation Kit LONG (TAKARA), following the manufacturer’s protocol. The recombinant plasmids were transformed into *Escherichia coli* strain DH10B by electroporation and plated on LB medium with kanamycin. Three recombinant plasmids containing (1) the entire genomic sequence of *Os04g0350700*, (2) the entire sequence of *Os04g0351333*, and (3) the coding region of *Os04g0351333* and its downstream noncoding region were obtained. To produce overexpression lines, the open reading frame (ORF) of *Os04g0350700* was obtained from the full-length cDNA of *Os04g0350700* using the SMARTer RACE cDNA Amplification kit (TAKARA) and cloned into *pENTR/D-TOPO* (Invitrogen). By LR recombination reaction (Invitrogen), the ORF was transferred into *pGWB502* and fused with the *CaMV35S* promoter (Nakagawa *et al.* 2007). All constructs were introduced into *Agrobacterium tumefaciens*, strain EHA105, by electroporation (Hood and Helmer 1986) and used to transform the awnless cultivar cv. Nipponbare following the methods of Hiei *et al.* (1994).

### RNA extraction and *RAE1* expression analysis

Immature lemmas were collected from panicles during the booting stage, whereas mature lemmas were collected from panicles during the heading stage of Koshihikari and GLSL-13. Total RNA was extracted from each sample using the RNeasy Plant Mini Kit (QIAGEN) and treated with RNase-Free DNase set (QIAGEN) to remove genomic DNA contamination. Omniscript RT Kit (QIAGEN) was used for first strand cDNA synthesis from each of the extracted RNA samples.

StepOne Real-Time PCR System (Applied Biosystems) was used to analyze the relative expression levels of *RAE1* in Koshihikari and GLSL-13. Reaction mixtures for real-time PCR were prepared following the manual of Power SYBR Green PCR Master Mix (Applied Biosystems). Relative expression levels were calculated by dividing *RAE1* expression levels by *UBQ* expression levels. The following primers were used for PCR of *RAE1* and *UBQ*: forward primer 5′-ATCCTCCTCTTCACGGCTTCTA-3′ and reverse primer 5′-CGTATGTACAGAAGGAGAGGTCG-3′ for *RAE1* and forward primer 5′-ACACGGTTCAACAACATCCA-3′ and reverse primer 5′-GATCAAGAACTAGAGCGTCA-3′ for *UBQ*.

### Data availability

The plant materials used in the present study are available upon request. Figure S1 shows the comparison of amino acid sequences of *RAE/An-1* between *O. glaberrima* and *O. sativa*. Table S1 provides the sequences of primers used in this study.

## Results

### Two loci cause awnless phenotype in *O. sativa*

**Figure 1:**
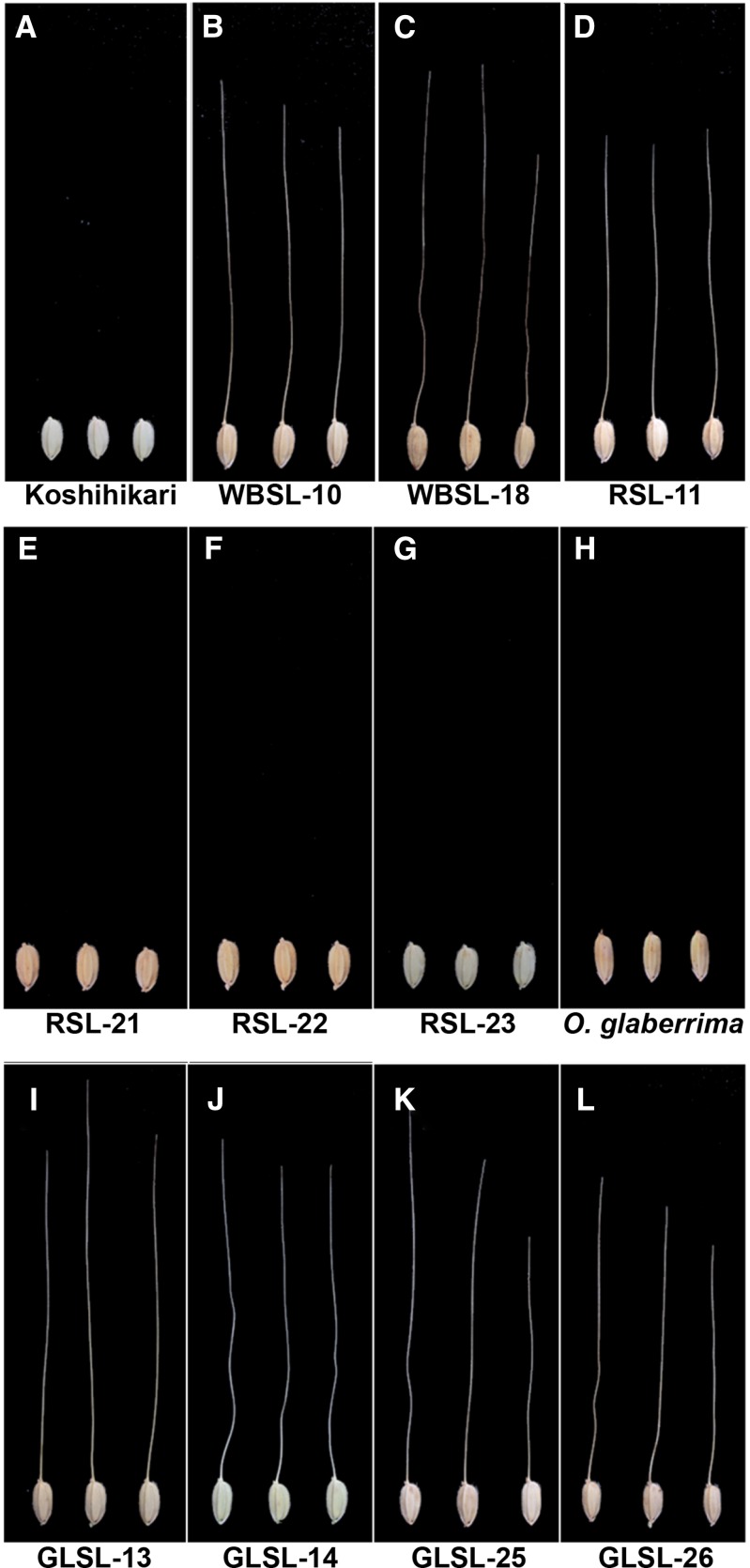
Long awn formation in CSSLs. Seed’s morphology of Koshihikari (A), WBSL-10 (B), WBSL-18 (C), RSL-11 (D), RSL-21(E), RSL-22 (F), RSL-23 (G), *O. glaberrima* (H), GLSL-13 (I), GLSL-14 (J), GLSL-25 (K), and GLSL-26 (L). Koshihikari and *O. glaberrima* do not form long awns. However, the CSSLs except for RSL-21, RSL-22, and RSL-23 have long awns at the distal end of lemmas. WBSLs: CSSLs harboring *O. nivara* chromosome segments in the genetic background of *O. sativa ssp. japonica* cv. Koshihikari. RSLs: CSSLs harboring *O. rufipogon* chromosome segments in the genetic background of *O. sativa ssp. japonica* cv. Koshihikari. GLSLs: CSSLs harboring *O. glaberrima* chromosome segments in the genetic background of *O. sativa ssp. japonica* cv. Koshihikari.

**Figure 2:**
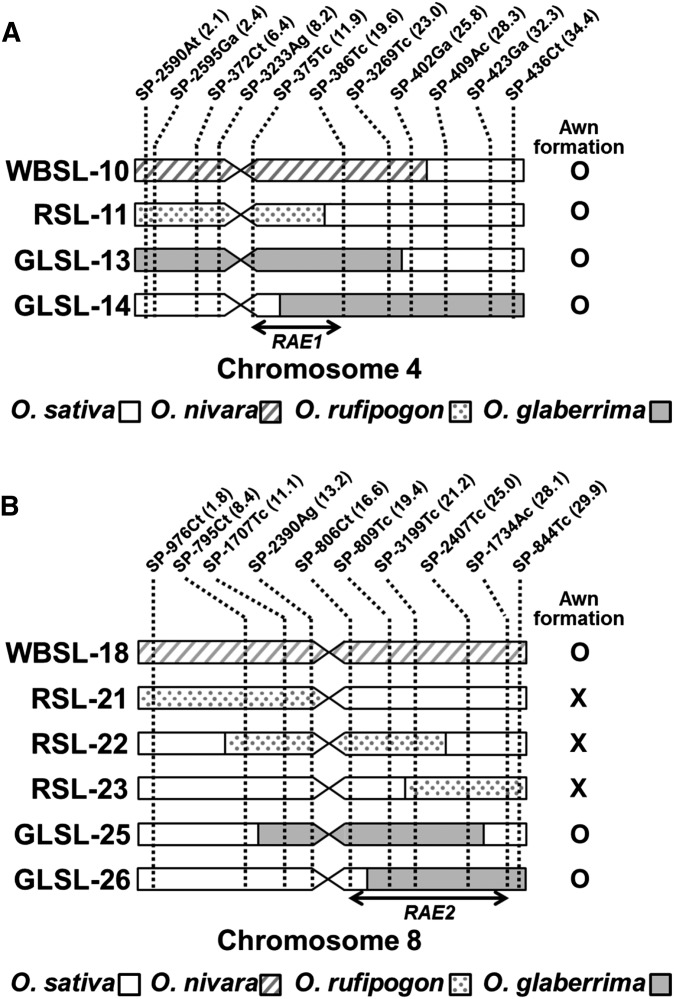
Graphical genotypes of the CSSLs forming long awns. Graphical genotypes of the CSSLs harboring substituted chromosome segments on chromosome 4 (A) and chromosome 8 (B). Chromosome segments of *O. sativa* cv. Koshihikari are represented by white boxes, whereas substituted segments from *O. nivara*, *O. rufipogon*, and *O. glaberrima* are indicated as shaded, dotted, and gray boxes, respectively. Presence and absence of long awn in each CSSL are indicated as O and X, respectively. Markers used for the development of the CSSLs are shown with their physical positions (Mb) (indicated in parenthesis) above the chromosome images. Candidate regions of *RAE1* and *RAE2* are indicated by double-headed arrows.

The CSSL population WBSLs were evaluated phenotypically for the presence of awns, and two awned lines were identified, WBSL-10 and WBSL-18 (Figure 1, A–C). Because the recurrent parent, Koshihikari, is an awnless variety, the formation of awns in WBSL-10 and WBSL-18 must be due to the substituted segments derived from *O. nivara*. WBSL-10 carries a ∼25-Mb donor segment on the end of the short arm of chromosome 4 (Figure 2A), whereas WBSL-18 has an entirely substituted chromosome 8 from *O. nivara* (Figure 2B). This result reveals that *O. nivara* contains two functional genes located on chromosomes 4 and 8, and either of which can independently induce awn formation in Koshihikari. Thus, we conclude that the functionality of at least two genes has been lost in *O. sativa*, resulting in an awnless phenotype. We named the causal genes on chromosomes 4 and 8, *RAE1* and *RAE2*, respectively.

To confirm our findings, we evaluated the awn phenotype in a second set of interspecific CSSLs, the RSLs. The RSL population also had a Koshihikari background but harbored *O. rufipogon* donor segments (Furuta *et al.* 2014). As expected, we found a long awn forming line, RSL-11 (Figure 1D). RSL-11 has a chromosome segment derived from *O. rufipogon* that spans ∼19 Mb on the end of the short arm of chromosome 4 (Figure 2A). However, in contrast to the results from the WBSL evaluation, we did not observe the induction of awns due to introgressions from the donor parent on chromosome 8: lines RSL-21, RSL-22, and RSL-23 were awnless, although they collectively possess donor segments covering the entire chromosome 8 (Figure 1, E–G; Figure 2B). This suggested a dysfunctional *RAE2* allele in the *O. rufipogon* donor. Evaluation of WBSLs and RSLs showed that *RAE1* and *RAE2* can independently induce the formation of long awns and that the awnless phenotype in *O. sativa* resulted from mutations in both *RAE1* and *RAE2*. A previous study by Luo *et al.* (2013) identified *An-1* in chromosome 4, a transcription factor that induces awn formation in rice. The candidate locus of *RAE1* identified in this study using *O. nivara* and *O. rufipogon* CSSLs includes the *An-1* locus, suggesting that *RAE1* and *An-1* might possibly be identical.

### Genetic independence of awnless phenotype in *O. sativa* and *O. glaberrima*

Most *O. sativa* and *O. glaberrima* varieties share the awnless phenotype (Figure 1, A and H), despite their independent domestication histories in Asia and Africa, respectively. To verify the functionality of *RAE1* and *RAE2* in *O. glaberrima*, we evaluated a third set of introgression lines, 34 GLSLs, for the presence of awns. GLSLs carry *O. glaberrima* chromosomal segments in a Koshihikari background (Shim *et al.* 2010). We identified four GLSLs with awns (GLSL-13, GLSL-14, GLSL-25, and GLSL-26) despite being derived from two awnless parents (Figure 1, A and I–L).

To investigate whether *RAE1* and *RAE2* were functional in these awned GLSLs, we examined the four lines for overlap with donor introgressions in the awned RSLs and WBSLs. We found a common 7.7-Mb region (11.9 Mb–19.6 Mb) on the proximal region of the long arm of chromosome 4 in WBSL-10, RSL-11, GLSL-13, and GLSL-14 (Figure 2A). We also discovered a shared donor region spanning 11.5 Mb in the awned lines WBSL-18, GLSL-25, and GLSL-26 on chromosome 8 (Figure 2B). These results implied that although the *O. glaberrima* donor is phenotypically awnless (Figure 1H), its genome harbors functional copies of *RAE1* and *RAE2* and is able to induce long awns in Koshihikari. At the same time, it is also implied that awnlessness in *O. glaberrima* arose from mutation(s) in gene(s) other than *RAE1* and *RAE2*. Our findings suggested that *O. sativa* and *O. glaberrima* have independently obtained the awnless phenotype through mutations in different genes.

### Fine mapping and identification of the causal gene for *RAE1*

**Figure 3:**
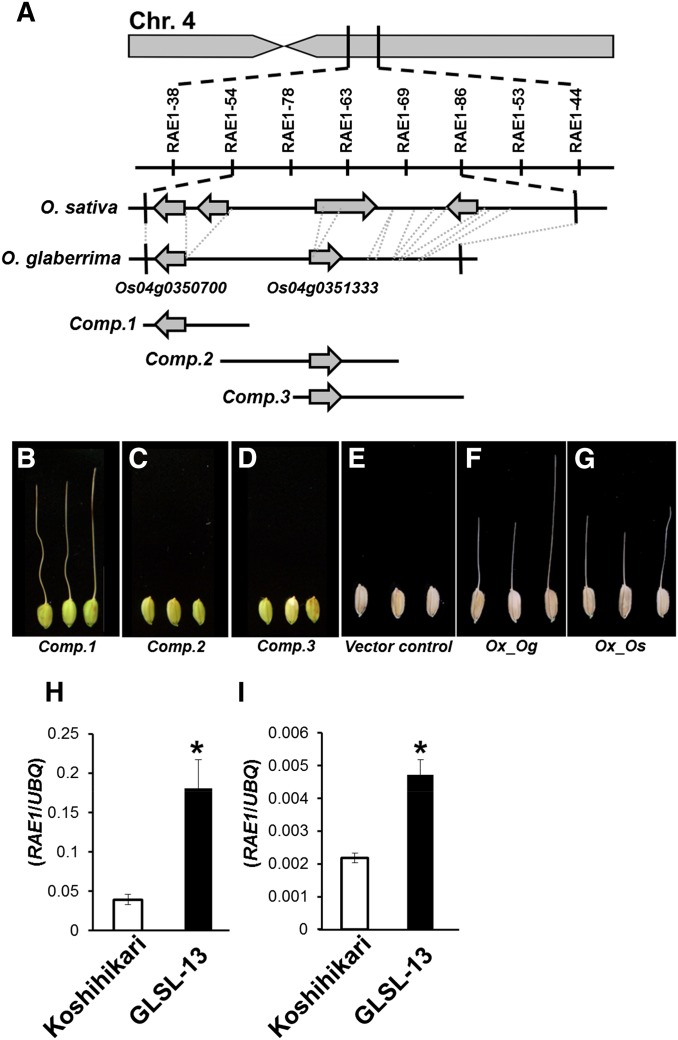
Positional cloning, complementation, and expression analysis of *RAE1*. (A) *RAE1* was mapped at the proximal part of the long arm of chromosome 4. This region has four annotated genes in *O. sativa*, whereas *O. glaberrima* only has two (*Os04g0350700* and *Os04g0351333*) of four genes in the corresponding region. (B–G) Complementation test and overexpression analysis were performed using awnless *O. sativa* cv. Nipponbare. Long awn formations were observed in the transgenic line of *Comp.1* harboring the entire genomic fragment of *Os04g0350700* derived from *O. glaberrima* (B), whereas *Comp.2* and *Comp.3* did not show awn formation (C and D). Overexpression of *O. glaberrima* allele (*Ox_Og*) (F) and Koshihikari allele (*Ox_Os*) (G) of *Os04g0350700* induced long awn formations in Nipponbare, whereas vector control showed no awn (E). (H and I) Expression analysis of *Os04g0350700* in immature lemma (H) and mature lemma (I) collected from GLSL-13 and Koshihikari. Mean values of three biological replicates are shown. An asterisk indicates the statistical significance at *P* < 0.01 in Student’s *t*-test.

Our study revealed that *O. glaberrima* also has functional *RAE1* on chromosome 4, even though this species do not form long awn. To clarify whether the causal gene for *RAE1 in O. glaberrima* is exactly identical to *An-1* or not, we performed fine mapping for *RAE1* in *O. glaberrima*. We used a population of ∼8000 F_2_ plants created from a cross between Koshihikari and GLSL-13, the introgression line containing a functional *RAE1* from *O. glaberrima*. We successfully mapped the *RAE1* location to a 60.4-kb region flanked by indel-based markers, RAE1-54 (16.73 Mb) and RAE1-86 (16.79 Mb), located on the long arm of chromosome 4 (Figure 3A). Sequence alignment revealed that the 60.4-kb candidate region for *RAE1* in *O. sativa* corresponded to a 41.1-kb region in *O. glaberrima*, which contained six large insertion/deletion regions. Our data showed that the *O. glaberrima* genome has only two candidate genes in this region, *Os04g0350700*, which was reported as *An-1*, and *Os04g0351333* (Figure 3A) (Luo *et al.* 2013).

To identify which of the two genes caused long awn formation, we performed a complementation test. The candidate region was subcloned from the BAC clone HWC026-A20 in three fragments, namely *Comp.1*, *Comp.2*, *and Comp.3* (Figure 3A). Of the three fragments, only *Comp.1* induced long awn formation in the transformants (Figure 3, B–D), implicating *Os04g0350700* as the causal gene for *RAE1*. Thus, these results verified that *RAE1* is identical to *An-1* and that the *O. glaberrima* allele of *RAE1/An-1* is functional for long awn formation.

*RAE1/An-1* has a 792-bp ORF and encodes 263 amino acids of a bHLH-type transcription factor of group 1A1, classified based on conserved motifs capable of binding E-box and G-box domains (Li *et al.* 2006) (Figure S1). We identified six SNPs and two indels in the *RAE1/An-1* coding region based on sequence comparison between *O. glaberrima* and *O. sativa* cv. Nipponbare (IRGSP-1.0). Although no polymorphism was located within the bHLH-conserved sequence, five of the eight mutations caused changes in the amino acid sequence (Figure S1). Additionally, the putative regulatory region upstream of the coding region in *O. sativa* contained a 4.4-kb transposable element (TE) insertion, along with numerous smaller indels, the same as with the case of *O. rufipogon* allele of *RAE1/An-1* described in the previous study for *An-1* (Luo *et al.* 2013).

Although sequence comparison between *O. glaberrima* and *O. sativa RAE1/An-1* alleles did not directly reveal which mutation(s) was responsible for the awnless phenotype in *O. sativa*, we hypothesized that the TE insertion in *O. sativa* might disrupt normal gene expression and performed additional experiments to test this. We produced overexpression lines of *RAE1/An-1* using alleles from both *O. glaberrima* and Koshihikari transformed into Nipponbare, a temperate *japonica* variety that harbors the same TE insertion found in Koshihikari. The overexpression lines of *RAE1/An-1* using both the *O. glaberrima* and Koshihikari alleles induced long awns in the Nipponbare background (Figure 3, E–G), proving that the Koshihikari *RAE1/An-1* indeed retains molecular function despite its awnless phenotype. Additionally, we compared *RAE1/An-1* expression in GLSL-13 and Koshihikari and found significantly increased expression in the introgression line *vs.* the recurrent parent in the immature and mature spikelet, respectively (Figure 3, H and I). These data confirm that it is an alteration of *RAE1/An-1* expression, rather than a structural change, that causes Koshihikari’s awnless phenotype. In the *An-1* work, the TE insertion at the promoter region of *An-1* has been suggested as the possible causal mutation for the loss of its expression (Luo *et al.* 2013). Therefore, the presence of the TE insertion in both Koshihikari and Nipponbare support that temperate *japonica* varieties of *O. sativa* might become awnless due to altered *RAE1 cis* regulation.

### Novel awn-inducing locus causes loss of long awns in *O. glaberrima*

**Figure 4:**
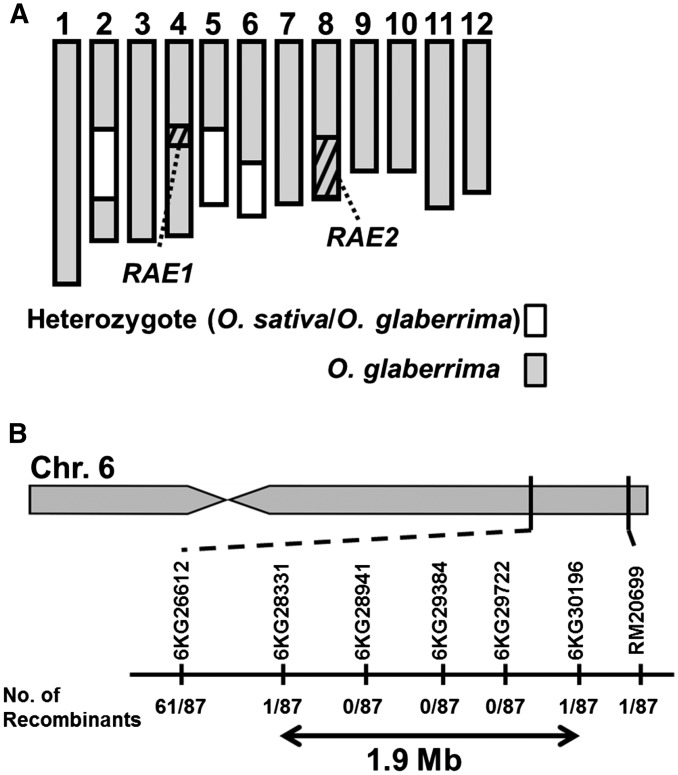
Location of causal locus for the loss of long awns in *O. glaberrima*. (A) The graphical genotype of the parental BC_4_F_1_ plant that showed segregation of long awn phenotype in its progenies. Gray boxes indicate homozygous regions derived from *O. glaberrima*, whereas heterozygous segments of *O. sativa* and *O. glaberrima* are indicated in white. Shaded boxes in chromosomes 4 and 8 represent the location of *RAE1/An-1* and the candidate region of *RAE2*, respectively. *RAE1* and *RAE2* regions have been fixed as the *O. glaberrima* homozygote in the BC_4_F_1_. (B) *RAE3* was roughly mapped at a 1.9-Mb region in chromosome 6 flanked by the genotype markers, 6KG28331 and 6KG30196. Numbers below the marker names indicate the number of recombinants.

Phenotypic analysis of the GLSLs showed that *O. glaberrima* had no long awns, despite retaining functional copies of both *RAE1* and *RAE2*. This finding suggested that another gene(s) was responsible for the loss of long awns in *O. glaberrima*. To identify this causal locus, we performed linkage analysis using progenies of the BC_4_F_1_ population derived from a cross between two awnless varieties: *O. sativa* cv. Taichung 65 × *O. glaberrima* (Acc IRGC104038), with *O. glaberrima* as the recurrent parent. In this BC_4_F_1_ population, we expect an average of 94.75% of the genome to be fixed for the *O. glaberrima* genotype and 6.25% to be heterozygous and able to segregate in the next generation. We evaluated progeny from 54 BC_4_F_1_ lines to identify families that were fixed for functional alleles at both *RAE1/An-1* and *RAE2* (*O. glaberrima* segments) but that still segregated phenotypically for long awns. We identified one such family of 87 BC_4_F_2_ plants that segregated phenotypically in a 3:1 ratio (61 long awned individuals *vs.* 26 awnless individuals). The parental BC_4_F_1_ plant of this population harbored heterozygous segments on chromosomes 2, 5, and 6 (Figure 4A).

We further analyzed the genotypes of these segments using the SSR markers RM341 (chromosome 2), RM6346 (chromosome 5), and RM20699 (chromosome 6). Among the 61 long awned individuals, RM341 and RM6346 allele frequencies were consistent with the theoretical segregation ratio for an F_2_ population; *P* values were 0.347 for RM341 and 0.975 for RM6346 in a chi-square test for 1:2:1 ratio (Table 1). In contrast, segregation of genotypes at RM20699 was highly distorted from the theoretical 1:2:1 ratio (*P* value = 2.94×10^−5^) (Table 1). Similar trends were observed in the 26 awnless plants (Table 1). From these results, we concluded that the genetic factor responsible for the awned phenotype in these BC_4_F_2_ plants was located at or near RM20699 on chromosome 6. The presence of a homozygous *O. glaberrima* segment around RM20699 eliminated awn formation, whereas heterozygous or homozygous *O. sativa* segments across that region induced long awn formation. Thus, a recessive allele found in *O. glaberrima* suppressed awn formation in this domesticated species, but the introduction of a functional *O. sativa* allele at this locus restored long awn formation. We named the causal gene on chromosome 6 regulating awn formation in *O. glaberrima RAE3*.

**Table 1:** Linkage between long awn phenotype and genotype

Segregation of Genotypes at the Three Markers in the Plants Forming Long Awn				
	*O. sativa*	Hetero	*O. glaberrima*	Total no. of plant
RM341 (Chr. 2)	11	31	19	61
RM6346 (Chr. 5)	15	30	16	61
RM20699 (Chr. 6)	18	43	0	61

Segregation of Genotypes at the Three Markers in the Plants Forming No Awn				
	*O. sativa*	Hetero	*O. glaberrima*	Total no. of plant
RM341 (Chr. 2)	4	16	6	26
RM6346 (Chr. 5)	4	10	12	26
RM20699 (Chr. 6)	0	1	25	26

Each number indicates the number of plants that are homozygous for the *O. sativa* allele and heterozygous or homozygous for the *O. glaberrima* allele at specific markers.

Further detailed mapping was performed using genotypic markers located around RM20699 (Figure 4B), which narrowed down the candidate region to 1.9 Mb (marker 6KG28331 to marker 6KG30196) (Figure 4B). Although fine mapping is required to identify the causal gene(s) underlying *RAE3*, our results indicate that we have identified a novel locus controlling long awn formation that is responsible for the loss of long awns in *O. glaberrima*, and that this locus is independent of the *RAE1/An-1* and *RAE2* loci controlling awn formation in *O. sativa*.

### Effects of awn formation on yield related traits

To investigate the effects of awn formation on yield related traits, we measured panicle length, number of primary branches, number of seeds, seed length, and seed width in Koshihikari and long-awned CSSLs (Table 2). We found that GLSL13, 14, and 26 showed a significant decrease in the number of seeds per panicle (Table 2). In GLSL-13, there was a significant increase of seed length, suggesting a trade-off between length and number of seeds. This result is consistent with the previous report for *An-1* (Luo *et al.* 2013). However, from our work, we did not observe a trade-off in number of seeds *vs.* seed length in CSSLs in two of the lines (GLSL-14 and GLSL-25). It is possible that other genes derived from the donor parents affected the traits we examined and masked the effects of long awn formation on yield-related traits. Interestingly, awn frequency per panicle exhibited significant differences among the CSSLs (Table 2). One striking example can be seen by comparing GLSL-13 with GLSL-14, where significantly different awn frequencies were observed despite the fact that both carry functional alleles at *RAE1/An-1*. This observation suggests the possibility that awn frequency is regulated independently from awn formation.

**Table 2:** Yield related traits and awn frequency per panicle in CSSLs

Lines	Panicle length (mm)	No. of primary branch	No. of seeds	Seed length (mm)	Seed width (mm)	Awn frequency per panicle^†^
Koshihikari	217.80±5.63	11.40±1.14	159.80±11.37	6.03±0.34	3.21±0.14	0.00±0.00^a^
RSL-11	200.20±13.54	10.80±0.84	147.40±12.05	6.05±0.36	3.19±0.16	64.43±15.40^b^
WBSL-10	200.00±12.81	9.80±0.45	146.60±12.42	6.04±0.32	3.11±0.14*	91.10±5.89^c^
WBSL-18	184.00±16.06*	13.60±1.82	167.80±15.74	6.22±0.28	3.04±0.12*	91.30±6.09^c^
GLSL-13	208.60±17.74	9.60±0.55	121.20±19.38*	6.59±0.32*	3.23±0.13	56.29±9.41^b^
GLSL-14	169.60±8.91*	10.60±2.07	110.80±18.57*	5.78±0.31*	3.10±0.13*	32.94±7.89^d^
GLSL-25	155.25±19.70*	9.00±0.82	68.50±22.49*	6.43±0.30*	3.27±0.11	92.34±2.67^c^
GLSL-26	193.80±6.53	12.60±1.14	141.20±7.09*	6.02±0.31	3.09±0.17*	83.62±6.17^c^

* P<0.05 in t-test with Holm’s adjustment comparing with Koshihikari.

† Mean ± sd followed by different letters indicate significantly different pairs detected in pairwise t-test with Holm’s adjustment (P<0.05).

## Discussion

The absence of long awns is one factor that helps distinguish the cultivated rice species, *O. sativa* and *O. glaberrima*, from their wild progenitors, *O. rufipogon* and *O. barthii*. While long, barbed awns serve to deter seed predation and enable seed dissemination in the wild, it is likely that they were actively selected against during domestication to facilitate harvesting and postharvest processing.

In this study, we demonstrate that an Asian rice cultivar, *O. sativa* cv. Koshihikari, is awnless due to two dysfunctional genes (*RAE1* and *RAE2*), whereas an African rice cultivar, *O. glaberrima* Acc IRGC104038, is awnless due to a third gene (*RAE3*). This discovery suggests that different awn loci were the targets of selection during the domestication of *O. sativa* and *O. glaberrima*, despite the phenotypic similarity. Previous studies have investigated genes involved in the domestication of *O. glaberrima* (Gross *et al.* 2010; Sanyal *et al.* 2010; Wang *et al.* 2014), but they focused on domestication genes that were already well-characterized in *O. sativa* (*e.g.*, *Rc*, *qSh1*, *Sd1*, *Dep1*, *OsSh1*, and *Sh4*) and concluded that convergent selection on common genes via independent mutations gave rise to similar phenotypes in Asian and African rice cultivars (Gross *et al.* 2010; Wang *et al.* 2014). In our study, we demonstrate that *O. sativa* and *O. glaberrima* have independently acquired the same phenotype, awnless seeds, via selection on different genes, namely *RAE1* and *RAE2* in *O. sativa* and *RAE3* in *O. glaberrima*. This is the first report showing that selection on independent mutations in different genes conferred the same phenotype in two closely related cultivated crop species.

Using our introgression lines, we found that introduction of either a functional *RAE1* or a functional *RAE2* allele could induce awn formation in cv. Koshihikari, a *temperate japonica* cultivar of *O. sativa*. This leads us to ask what phenotype might have been under selection by humans during the domestication of *O. sativa* if selection for a dysfunctional allele at only one of the loci already produces awnless rice. One possible explanation is that *RAE1* and *RAE2* have pleiotropic effects on other biological processes that may have been the targets of selection during domestication. Luo *et al.* (2013) reported pleiotropic effects of the awn gene, *An-1* (equivalent to *RAE1* in this study), using NILs in an *indica* background. In that study, NIL-An-1, containing a functional *An-1* allele derived from *O. rufipogon*, exhibited a reduction in seed number per panicle and increased seed length compared with the *indica* recurrent parent, Guangluai4. Although significant, the difference in seed number between NIL-An-1 and Guangluai4 was only an average of 10 seeds per panicle. In our study, we observed trade-offs between seed length and seed number in one CSSL, whereas others showed no correlation between seed length and seed number.

Another explanation is that both functional and dysfunctional *RAE2* alleles already existed as standing variation in the wild, making it likely that *RAE1* was the target of direct selection during domestication (in at least one subpopulation of *O. sativa* whose ancestor carried a functional allele of *RAE2*). Subsequent recombination would easily have brought *RAE1* and *RAE2* together. We see hints of this in the wild donors of the CSSLs used in this study; the *O. nivara* parent used in the WBSL evaluation carried a functional allele at *RAE2*, whereas the *O. rufipogon* parent that was used in the RSL evaluation did not. More detailed genome-wide analyses using diverse cultivars and wild relatives will be required to determine the frequencies of both *RAE1* and *RAE2* alleles in *O. sativa* and its wild relatives, and will allow us to explore hypotheses about which gene was selected on first and unveil the processes leading to the awnless phenotype during domestication of *O. sativa*.

Our finding that a mutation(s) in *RAE3* causes the loss of long awns in *O. glaberrima*, which carries functional alleles of both *RAE1* and *RAE2*, suggests that *RAE3* functions as a common upstream or downstream factor of both *RAE1* and *RAE2*, interacting epistatically with *RAE1* and *RAE2*. Evaluation of GLSL-13 and GLSL-14 demonstrated that the introduction of functional alleles of *RAE1* can induce long awns in a genotype carrying dysfunctional alleles at *RAE2*, and evaluation of GLSL-25 and GLSL-26 demonstrated that functional alleles at *RAE2* in combination with dysfunctional alleles at *RAE1* also induces long awn formation. In *O. glaberrima*, despite the presence of functional alleles at both *RAE1* and *RAE2*, the long awned phenotype was eliminated by a mutation in *RAE3*. Isolation and molecular characterization of *RAE3* will open the door to a better understanding of the regulatory mechanism(s) underlying long awn formation in rice.

Previous genetic studies identified five loci associated with awn formation. These included: (1) *An8*, located on the long arm of chromosome 4; (2) *An6*, located on the long arm of chromosome 8; (3) *An7* located on the short arm of chromosome 5; (4) *An9* located on the short arm of chromosome 1; and (5) *An10* on the long arm of chromosome 1 (Yoshimura *et al.* 2010). All were identified using introgression lines harboring genomic segments derived from *Oryza glumaepatula* and *Oryza meridionalis. An8* colocates on chromosome 4 with *RAE1/An-1*, and *An6* colocates on chromosome 8 with *RAE2*, reported here. In the genetic material used in our study, we did not observe long awns in the CSSLs harboring substituted segments in the regions corresponding to *An7*, *An9*, and *An10*. It is possible that *O. glumaepatula* and *O. meridionalis* have functional alleles of *An7*, *An9*, and *An10*, whereas *O. sativa*, *O. rufipogon*, *O. nivara*, and *O. glaberrima* have lost them.

Our observations about differing frequencies of awns on panicles of the CSSLs support the hypothesis that multiple genes affect awn development in rice. Thus, we conclude that awn formation is regulated by a complex network of interacting genes, despite its simple morphology. As a phenotype, awn formation offers an interesting model for studying plant morphogenesis and development, as well as crop evolution and domestication. Further in-depth molecular analysis will be required to unravel the fine-tuned genetic control and pleiotropic impacts of awn development in rice.

## 

## Supplementary Material

Supporting Information
