# Supplementary material for: Convergent Loss of Awn in Two Cultivated Rice Species Oryza sativa and Oryza glaberrima Is Caused by Mutations in Different Loci
Source: G3 (Bethesda). 2015 Sep 2;5(11):2267–74. doi: 10.1534/g3.115.020834 (PMC4632046; doi:10.1534/g3.115.020834)
Supplement: Supporting Information [file supp_5_11_2267__index.html]

Convergent Loss of Awn in Two Cultivated Rice Species Oryza sativa and Oryza glaberrima Is Caused by Mutations in Different Loci — Supporting Information 

# Convergent Loss of Awn in Two Cultivated Rice Species *Oryza sativa* and *Oryza glaberrima* Is Caused by Mutations in Different Loci

## Supporting Information for Furuta *et al.*, 2015

**Files in this Data Supplement:**

- Supporting Information - Figure S1 and Table S1 (PDF, 217 KB)
- Figure S1 - Amino acid sequences of *RAE1/An-1* in *O. glabeerima* and *O. sativa*. (PDF, 212 KB)
- Table S1 - Primers used in this study. (PDF, 83 KB)
