# Supplementary material for: Convergent Loss of Awn in Two Cultivated Rice Species Oryza sativa and Oryza glaberrima Is Caused by Mutations in Different Loci
Source: G3 (Bethesda). 2015 Sep 2;5(11):2267–74. doi: 10.1534/g3.115.020834 (PMC4632046; doi:10.1534/g3.115.020834)
Supplement: Supporting Information [file supp_g3.115.020834_020834SI.pdf]

G3 · Genes | Genomes | Genetics

Convergent loss of awn in two cultivated rice species *Oryza sativa* and *Oryza glaberrima* is caused by mutations in different loci

Tomoyuki Furuta[*], Norio Komeda[*], Kenji Asano[*], Kanako Uehara[*], Rico Gamuyao[*], Rosalyn B. Shim-Angeles[*], Keisuke Nagai[*], Kazuyuki Doi[*], Diane R. Wang[§], Hideshi Yasui[†], Atsushi Yoshimura[†], Jianzhong Wu[‡], Susan R. McCouch[§] and Motoyuki Ashikari[*]

[*] Bioscience and Biotechnology Center, Nagoya University, Furo-cho, Chikusa, Nagoya, Aichi 464-8601, Japan

[§] Department of Plant Breeding and Genetics, Cornell University, 162 Emerson Hall, Ithaca, NY 14853-1901, USA

[†] Plant Breeding Laboratory, Kyushu University, 6-10-1 Hakozaki, Higashi-ku, Fukuoka 812-8581, Japan

[‡] National Institute of Agrobiological Sciences, Tsukuba, Ibaraki 305-8634, Japan

Corresponding author: Susan R. McCouch and Motoyuki Ashikari

For Susan R. McCouch

Address: Department of Plant Breeding and Genetics, Cornell University, 162 Emerson Hall, Ithaca, NY 14853-1901, USA

TEL: 1-607-255-0420

Email: srm4@cornell.edu

For Motoyuki Ashikari

Address: Bioscience and Biotechnology Center, Nagoya University, Furo-cho, Chikusa, Nagoya, Aichi 464-8601, Japan

TEL: 81-52-789-5202

Email: ashi@agr.nagoya-u.ac.jp

DOI: 10.1534/g3.115.020834

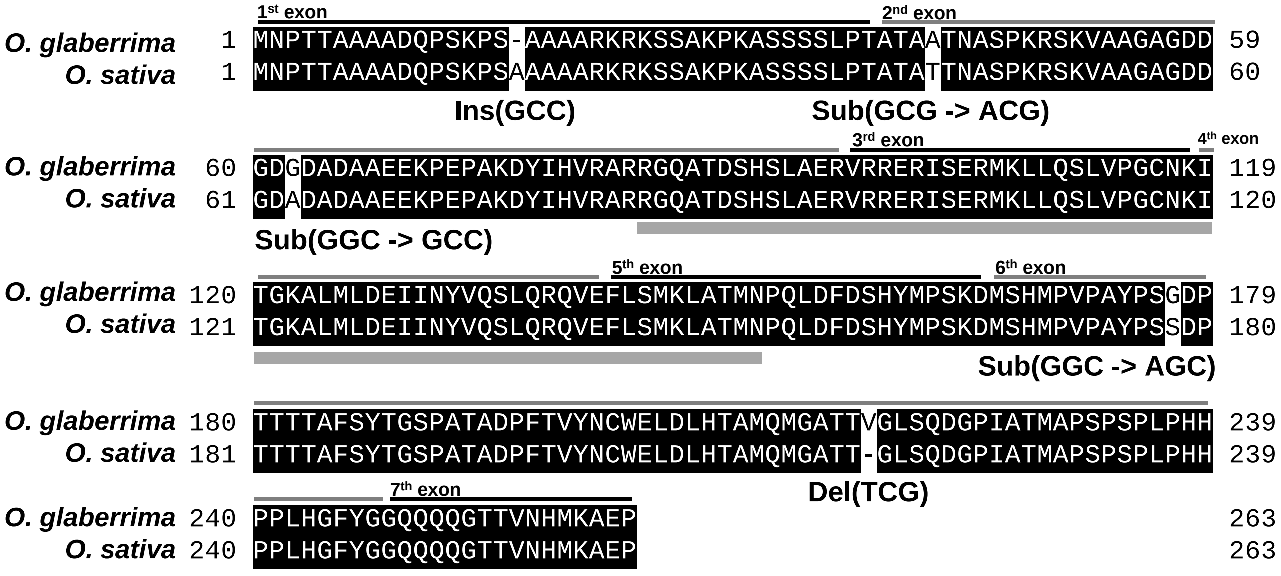

Figure S1 Amino acid sequences of *RAE1/An-1* in *O. glabeerima* and *O. sativa*

Amino acid sequences of *RAE1/An-1* in *O. glaberrima* and *O. sativa* are aligned by ClustalW. Mutations in the DNA sequence are shown below the mutation sites. The thick gray bar under the aligned sequences represents a conserved bHLH domain. The thin black bars and gray bars above the aligned sequences indicate the region of exons.

Table S1 Primers used in this study

Primers for linkage analysis of *RAE1*		
RAE1-38F	AGATTGGTTTAGGCTGCAGG	SSR
RAE1-38R	AAGGGCAGCAAATCAGGTAC	
RAE1-54F	TCACTAGCACAATCCTCCTC	SSR
RAE1-54R	GGCAAAGAACTTGTTTTGATGTTGTGAAG	
RAE1-78F	TTCTAGCACTACTAGAAAACACGTTTCTGC	dCAPS(PstI)
RAE1-78R	ATATTTTTGCAGGCGGGTGG	
RAE1-63F	GCTACAGTCCACGCCGCAACCCTCCGGTAC	dCAPS(KpnI)
RAE1-63R	CGTGCATGTTTTAATCCGCC	
RAE1-69F	GAAGGGCAGGAAAACAAGAG	SSR
RAE1-69R	TTGTGTCCGGTGAGATTTGG	
RAE1-86F	TGTATGAAATGTTACAGAACTTGCTCTCGA	dCAPS(XhoI)
RAE1-86R	GTCATATGCGTGCACTTGAC	
RAE1-53F	TTGGAAAACACAGTGCCGTC	SSR
RAE1-53R	CGATGGAGCAGTAGTTAGAG	
RAE1-44F	AGCGTTCAACGTTTGGATGG	SSR
RAE1-44R	TCAGAGACAGAGTCAAGTGC	
Primers for linkage analysis of *RAE3*		
RM341-F	CAAGAAACCTCAATCCGAGC	SSR
RM341-R	CTCCTCCCGATCCCAATC	
RM6346-F	ACTTTGATCGATCAGCCACC	SSR
RM6346-R	AGGTGGTGGAGATGAAGCAG	
RM20699-F	CCCGAGCCAGACAACATTCC	SSR
RM20699-R	GAGGTGTGAGGTGAGGAAGATGC	
6KG27612-F	TAGGTAGGAGTAGGCCGGAT	Indel
6KG27612-R	GCATGCACATATGTCACTGTGTAA	
6KG28331-F	CGATCTCCTTTGCATCTTTC	Indel
6KG28331-R	GGTGGTTAGCACCTTTGTGT	
6KG28941-F	CTCCTCTGATCACCTCGCT	Indel
6KG28941-R	GGAGAGGAGCAGCTTCTTG	
6KG29384-F	GTTTTGCTCAGGCAAATGAT	Indel
6KG29384-R	GCACCCAAGATTTTATTGGA	
6KG29722-F	ATGTTAGCCTTTTTCCTCCA	Indel
6KG29722-R	GGTCTTCGTTAGTCTTATGCATCT	
6KG30196-F	CTTGTTTCCATTTTGTTGGG	Indel
6KG30196-R	GGAGGAAGAAGAGGACGAAG	
