# Supplementary material for: Convergent Loss of Awn in Two Cultivated Rice Species Oryza sativa and Oryza glaberrima Is Caused by Mutations in Different Loci
Source: G3 (Bethesda). 2015 Sep 2;5(11):2267–74. doi: 10.1534/g3.115.020834 (PMC4632046; doi:10.1534/g3.115.020834)
Supplement: Supporting Information [file supp_g3.115.020834_FigureS1.pdf]

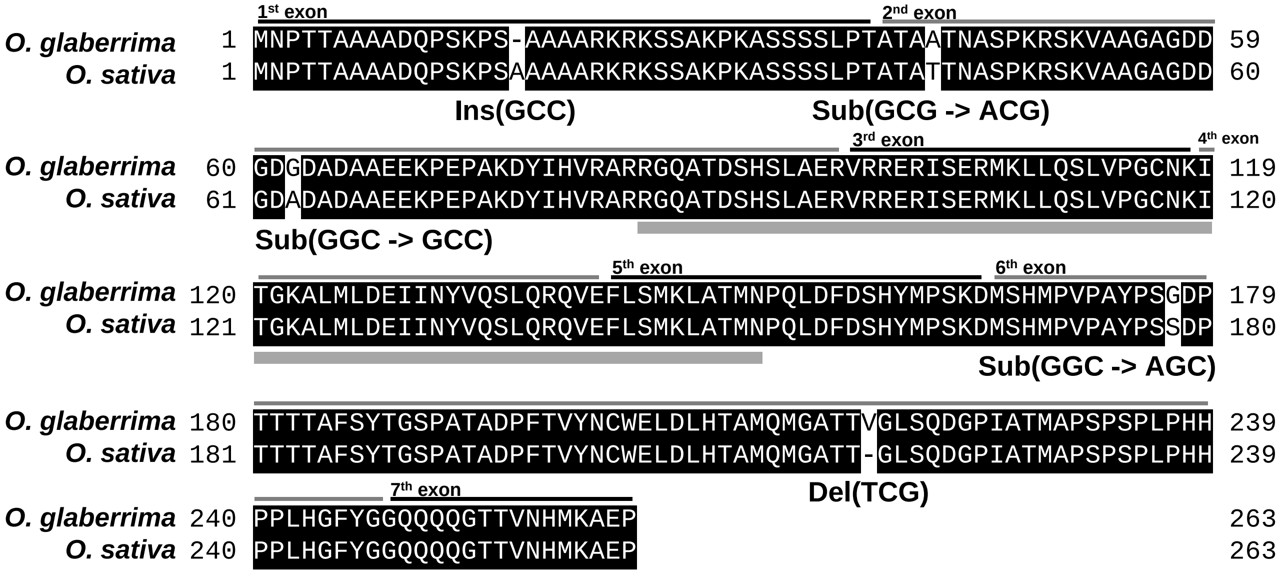

Figure S1 Amino acid sequences of *RAE1/An-1* in *O. glabeerima* and *O. sativa*
Amino acid sequences of *RAE1/An-1* in *O. glaberrima* and *O. sativa* are aligned by ClustalW. Mutations in the DNA sequence are shown below the mutation sites. The thick gray bar under the aligned sequences represents a conserved bHLH domain. The thin black bars and gray bars above the aligned sequences indicate the region of exons.
