# Supplementary material for: Convergent Loss of Awn in Two Cultivated Rice Species Oryza sativa and Oryza glaberrima Is Caused by Mutations in Different Loci
Source: G3 (Bethesda). 2015 Sep 2;5(11):2267–74. doi: 10.1534/g3.115.020834 (PMC4632046; doi:10.1534/g3.115.020834)
Supplement: Supporting Information [file supp_g3.115.020834_TableS1.pdf]

Table S1 Primers used in this study

Primers for linkage analysis of *RAE1*		
RAE1-38F	AGATTGGTTTAGGCTGCAGG	SSR
RAE1-38R	AAGGGCAGCAAATCAGGTAC	
RAE1-54F	TCACTAGCACAATCCTCCTC	SSR
RAE1-54R	GGCAAAGAACTTGTTTTGATGTTGTGAAG	
RAE1-78F	TTCTAGCACTACTAGAAAACACGTTTCTGC	dCAPS(PstI)
RAE1-78R	ATATTTTTGCAGGCGGGTGG	
RAE1-63F	GCTACAGTCCACGCCGCAACCCTCCGGTAC	dCAPS(KpnI)
RAE1-63R	CGTGCATGTTTTAATCCGCC	
RAE1-69F	GAAGGGCAGGAAAACAAGAG	SSR
RAE1-69R	TTGTGTCCGGTGAGATTTGG	
RAE1-86F	TGTATGAAATGTTACAGAACTTGCTCTCGA	dCAPS(XhoI)
RAE1-86R	GTCATATGCGTGCACTTGAC	
RAE1-53F	TTGGAAAACACAGTGCCGTC	SSR
RAE1-53R	CGATGGAGCAGTAGTTAGAG	
RAE1-44F	AGCGTTCAACGTTTGGATGG	SSR
RAE1-44R	TCAGAGACAGAGTCAAGTGC	
Primers for linkage analysis of *RAE3*		
RM341-F	CAAGAAACCTCAATCCGAGC	SSR
RM341-R	CTCCTCCCGATCCCAATC	
RM6346-F	ACTTTGATCGATCAGCCACC	SSR
RM6346-R	AGGTGGTGGAGATGAAGCAG	
RM20699-F	CCCGAGCCAGACAACATTCC	SSR
RM20699-R	GAGGTGTGAGGTGAGGAAGATGC	
6KG27612-F	TAGGTAGGAGTAGGCCGGAT	Indel
6KG27612-R	GCATGCACATATGTCACTGTGTAA	
6KG28331-F	CGATCTCCTTTGCATCTTTC	Indel
6KG28331-R	GGTGGTTAGCACCTTTGTGT	
6KG28941-F	CTCCTCTGATCACCTCGCT	Indel
6KG28941-R	GGAGAGGAGCAGCTTCTTG	
6KG29384-F	GTTTTGCTCAGGCAAATGAT	Indel
6KG29384-R	GCACCCAAGATTTTATTGGA	
6KG29722-F	ATGTTAGCCTTTTTCCTCCA	Indel
6KG29722-R	GGTCTTCGTTAGTCTTATGCATCT	
6KG30196-F	CTTGTTTCCATTTTGTTGGG	Indel
6KG30196-R	GGAGGAAGAAGAGGACGAAG	
